# Respiratory motion tracking system of hepatocellular carcinoma treatment using FUS

**DOI:** 10.1186/2050-5736-3-S1-P47

**Published:** 2015-06-30

**Authors:** Hiroyuki Fukuda, Nobutaka Doba, Kazushi Numata, Ayako Takeda, Yoshiharu Hao, Akito Nozaki, Masaaki Kondo, Makoto Chuma, Shin Maeda, Tatsuya Fujii, Dongjuin Lee, Norihiro Koizumi, Hiroyuki Tsukihara, Mamoru Mitsuishi, Yoichiro Matsumoto, Kiyoshi Yoshinaka, Katsuaki Tanaka

**Affiliations:** 1 Yokohama City University, Yokohama, Japan; 2 The University of Tokyo, Tokyo, Japan; 3 National Institute of Advanced Industrial Science and Technology, Tsukuba, Japan

## Background/introduction

One of the reasons for the long treatment time of FUS for HCC compared with RFA is that we have to adjust the target lesion which has a respiratory movement. In this study, we evaluated the usefulness of respiratory tracking system for the FUS monitoring images of hepatocellular carcinoma (HCC).

## Methods

The respiratory motion trackings for the focal lesions were performed in 6 patients with HCC. The maximum diameter of the tumors measured on sonography ranged from 10 to 50 mm (mean, 25 mm; SD, 5.5 mm). The FUS system (Mianyong Haifu Tech) was used under ultrasound guidance. By using the video images during the FUS treatment, we evaluated the respiratory motion tracking system retrospectively. Template matching method was applied to this respiratory motion tracking software.

## Results and conclusions

**Figure 2:**
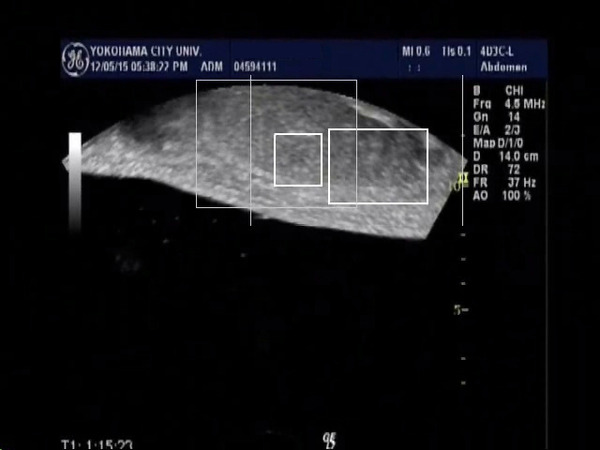
Tumor of the monitor ultrasonography in breath in position

**Figure 3:**
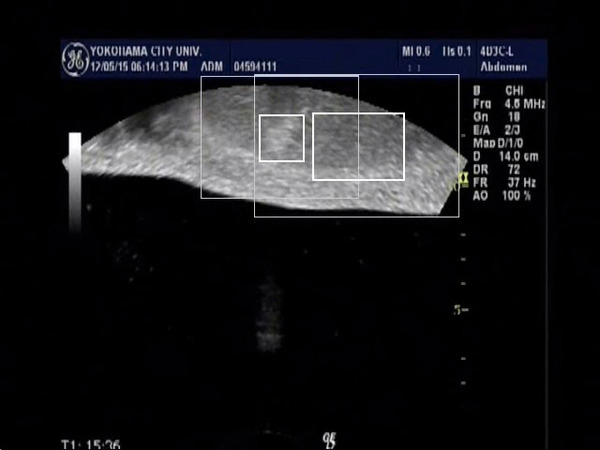
Tumor surrounded with hyperecho after the FUS treatment was also tracked successfully in both breath out position

**Figure 4:**
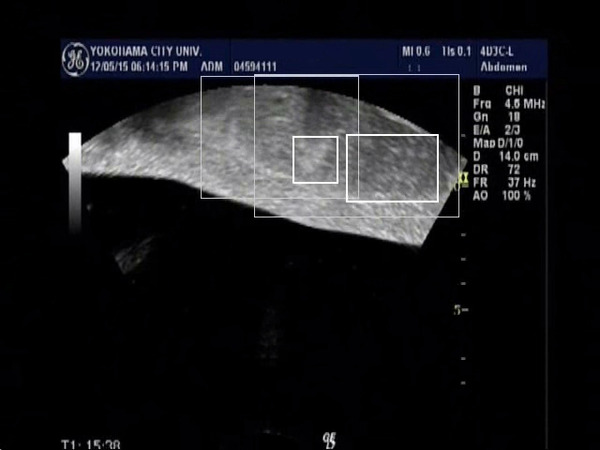
Tumor surrounded with hyperecho after the FUS treatment in both breath in position

In 6 cases of HCC, we evaluated the tracking system according to the tumor size, and the tracking of the tumor larger 3cm in diameter were successfully performed in all cases (n=2). On the other hand, the tracking of the tumor smaller than 3cm were performed successfully in 3 out of 4 cases. The reason for motion trackings were not performed well was thought to be that the tumor contour became unclear because the maximal cross section of the tumor go out of the plane by the respiratory movement. By using the video images during the FUS treatment (Fig. 1, Fig. 2), we evaluated the respiratory tracking, and the tumor was successfully tracked. And the tumor after FUS treatment was also tracked well even after the presence of the hyperecho around the tumor (Fig. 3, Fig. 4). In conclusion, the respiratory motion tracking using template matching method was successfully performed and have a possibility to shorten the FUS treatment time.

**1:**
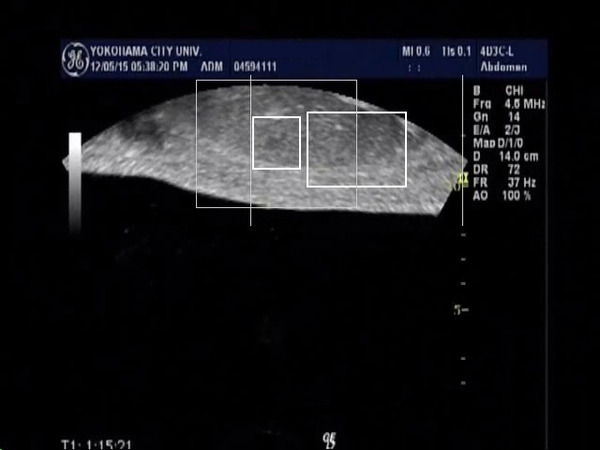
Tumor of the monitor ultrasonography was tracked in the small square, and it was tracked successfully in breath out position

